# Editorial: Metabolism in nonalcoholic fatty liver disease

**DOI:** 10.3389/fphys.2023.1275319

**Published:** 2023-08-22

**Authors:** Sonia Michael Najjar, Hilda Elias Ghadieh, Revathi Sekar, Raffaele Carraro, Lilia G. Noriega, Antonio Marcus de Andrade Paes

**Affiliations:** ^1^ Department of Biomedical Sciences, Heritage College of Osteopathic Medicine, Ohio University, Athens, OH, United States; ^2^ Diabetes Institute, Heritage College of Osteopathic Medicine, Ohio University, Athens, OH, United States; ^3^ Department of Biomedical Sciences, Faculty of Medicine and Medical Sciences, University of Balamand, Kalhat Al Koura, Tripoli, Lebanon; ^4^ Institute for Diabetes and Cancer, Helmholtz Munich, Neuherberg, Germany; ^5^ Department of Endocrinology, University Hospital La Princesa, Madrid, Spain; ^6^ Department of the Physiology of Nutrition, National Institute of Medical Sciences and Nutrition Salvador Zubirán, Mexico City, Mexico; ^7^ Department of Physiological Sciences, Biological and Health Sciences Center, Federal University of Maranhão, São Luís, Brazil

**Keywords:** NAFLD (non alcoholic fatty liver disease), insulin resistance, prediabetes, prevent and control, racial and ethnic differences

Non-alcoholic fatty liver (NAFLD), recently renamed metabolic dysfunction-associated fatty liver disease (MAFLD) and steatotic liver disease (SLD) is reaching a global epidemic, affecting ∼24% of the world population. Some ethnic groups, such as South Americans and Middle-Easterns are at a high risk to develop this disease (Younossi et al., 2018). Although some patients are lean, NAFLD is commonly associated with obesity and type 2 diabetes, two metabolic abnormalities that are marked by insulin resistance. This has provided the impetus to repurpose insulin sensitizers and incretins for NAFLD treatment (Gastaldelli and Cusi, 2019). However, whether insulin resistance underlies the broad manifest of NAFLD is still under debate and other metabolism-related risk factors are considered, as highlighted in the studies included in this Research Topic.

Shaheen et al. analyzed data of 3,190 adults from the National Health and Nutrition Examination Survey (NHANES) 2017–2018. They identified prediabetes as a growing risk factor for moderate-to-severe NAFLD and HbA1c as an independent predictor of NAFLD severity. These associations are stronger in Mexican American males who are at higher risk of developing severe NAFLD relative to other racial/ethnic groups (*p* < 0.05). This study emphasized the strong association between deranged insulin action and predisposition to NAFLD and indicated that screening for NAFLD should begin in patients with prediabetes before overt diabetes develops.

Mátis et al. presented a summary of longitudinal studies evaluating the association between long-term improvement in body composition and NAFLD reversal. They found that reducing visceral (VAT) and subcutaneous adipose tissue (SAT) mass and myosteatosis in addition to increasing skeletal muscle lean mass are associated with reversal of hepatic steatosis. Although randomized controlled trials focusing on body composition in patients with NAFLD are needed to further evaluate this association, the review suggests that maintaining functionally healthy muscle mass and reducing VAT and SAT prevent hepatic steatosis.

The contribution of childhood obesity to NAFLD in adulthood is well established and the incidence of NAFLD in children in the US has increased substantially (Younossi et al., 2018). The nutritional and hormonal microenvironment pre-and postnatally play a critical role in disease outcome (Rinaudo and Wang, 2012). Thus, de Souza et al., explored whether elevated glucocorticoid levels mediate the positive effect of childhood obesity on NAFLD risk in adulthood. To this end, they used litter size reduction of Wistar rats at 3 days of age to cause lactation overnutrition and obesity in the few remaining male rats. In addition to hepatic steatosis, elevated plasma triglycerides (TG) and high-density lipoprotein cholesterol (HDL-c), overfed male rats manifested high plasma glucocorticoids levels. Adrenalectomy (ADX) at 60 days of age attenuated the metabolic abnormalities, while glucocorticoids treatment of ADX rats restored hepatic steatosis and hyperlipidemia compared with the sham-treated group. This demonstrated the permissive role of glucocorticoids in maintaining hyperlipidemia and NAFLD in adulthood.

Whereas the above studies analyzed the importance of obesity and body fat distribution in NAFLD pathogenesis, Li et al. focused on NAFLD risk in non-obese Chinese adults. They carried out a 5-year-secondary prospective cohort study on 11,891 non-obese Chinese adult volunteers with 54.68% being males. During a median follow-up of 29.35 months, 17.05% of the participants were diagnosed with NAFLD. These subjects manifested a ratio of gamma glutamyl transferase (GGT) to HDL-c (GGT/HDL-c) of <20.35 and normal levels of low-density lipoprotein cholesterol (LDL-c). This positive and non-linear relationship between NAFLD and GGT/HDL-c ratio was found to be stronger in adults with TG < 1.7 mmol/L. The results suggest that maintaining GGT/HDL-c <20.35 prevents NAFLD, particularly in lean adults.

Rohbeck et al. focused on the role of GABA receptor in NAFLD pathogenesis since GABA protects the liver against injury. To this end, they treated HepG2 human hepatoma cells with palmitate (to induce lipotoxicity) in the absence and presence of HK4, a novel positive allosteric modulator of the GABA_A_ receptor. HK4 was shown to specifically target mitochondrial respiration, protein ubiquitination, apoptosis and cell cycle as well as transcription factors responsible for DNA repair, cell cycle progression and ER stress to restore the initial gene expression pattern of healthy hepatocytes. The study proposed that HK4 may represent an innovative pharmacological tool to treat or prevent NAFLD.

Further delving into identifying risk factors of NAFLD independent of obesity and metabolic alterations, Ma et al. conducted a meta-analysis that included 25 studies involving 107,306 participants to further examine the relationship between *Helicobacter Pylori* (*H. Pylori*) infection and NAFLD. Their review showed an increased risk of NAFLD in Asian females with *H. Pylori* infection but not in Asian males and that this observation was consistent among the various studies despite differences in NAFLD diagnosis methods and study design. Whereas non-Asian females and males with *H. Pylori* infection showed a higher risk for NAFLD, this association was not stable enough according to subgroup and sensitivity analyses. While these studies should be expanded to yield a more stable association in non-Asian populations, the review presented an intriguing hypothesis that *H. Pylori* infection may constitute a novel mechanistic link to NAFLD, especially in Asian women.

To date, there is no approved pharmacological therapy targeting NAFLD. In this Research Topic, Rohbeck et al. carried out the very early steps in demonstrating the effectiveness of HK4 in preventing lipotoxicity in palmitate-treated HepG2 cells. While the evaluation of the efficacy of this and other approaches in NAFLD-targeted therapy awaits long-term *in vivo* studies, preventive means should be adapted to curb the spread of the disease and its co-morbidities. Studies by Shaheen et al. showed that maintaining glycemic control in subjects with pre-diabetes is effective in preventing NAFLD. Mátis et al. proposed the design of special diets and exercise programs that reduce abdominal adipose mass while increasing lean muscle mass to resolve NAFLD effectively. de Souza et al. recommended the lowering of plasma glucocorticoids levels to prevent NAFLD progression. Li et al. recommended maintaining a GGT/HDL-c ratio lower than 20.35 to prevent NAFLD, particularly in lean Chinese adults. Finally, Ma et al. proposed eradicating *H. Pylori* infection as a novel preventive approach against NAFLD development in Asian women. In summary, this Research Topic provides a platform on which to discuss the design and implementation of tools to prevent NAFLD development in various populations.
